# Supplementary material for: Prevalence and risk factors of coliform-associated mastitis and antibiotic resistance of coliforms from lactating dairy cows in North West Cameroon
Source: PLoS One. 2022 Jul 26;17(7):e0268247. doi: 10.1371/journal.pone.0268247 (PMC9321367; doi:10.1371/journal.pone.0268247)
Supplement: Attachment — Submitted filename: Coliform mastitis paper Cameroon.docx [file pone.0268247.s001.docx]

Review of:

**Prevalence, Risk Factors and Antibiotic Resistance of Coliforms from Lactating Dairy Mastitis Cows in North West Cameroon**

Comments:

L 1 The title of this manuscript is misleading. Clinical and subclinical infections of the mammary glands differ; whereas milk from cows with **clinical** **mastitis** is not approved for sale or consumption, but cows with **subclinical mastitis** may have their milk sold or consumed, depending on the Somatic Cell Count, which differs among countries. For example, in the EU, the SCC of milk for sale cannot exceed 400,000 over a 3-month period in herds that must be sampled at least once monthly. A more accurate title would simply delete the word “Mastitis” and discuss it in appropriate context in the manuscript.

L30 California Mastitis Test instead of California mastitis Test

L35 It is important here to define what is meant by the term “mastitis”. In this study, cows with active coliform organisms in their milk were classified as having mastitis, regardless of their SCC. Some of these cows would have met the European standards for salable milk. This needs to be clarified.

L37 It would be more correct to say that these cows had Coliform infections, rather than saying Coliform mastitis.

L81-82 This statement is incorrect. If only a few Coliform organisms were detected in milk from a quarter, then this would not be considered mastitis. It would be more correct to say that about 70-80% of clinical mastitis cases are associated with Coliform infections.

L88 There are several methods to treat Coliform infections instead of using antibiotics. For example, frequent milking to expel the infected milk, sometimes with use of oxytocin, can eliminate the infection without antibiotics.

L91-93 Transmission through the food chain can be reduced greatly by pasteurization of the milk.

L93-94 Monitoring resistance has no effect on control of mastitis unless cows with resistant Coliforms are culled.

L115 See this similar paper from Cameroon: <https://www.heraldopenaccess.us/openaccess/bacterial-pathogens-involved-in-bovine-mastitis-and-their-antibiotic-resistance-patterns-in-the-adamawa-region-of-cameroon> This paper may be from a different region and it differs in some respects from the current paper, but it clearly shows the causes of clinical mastitis in Cameroon.

L136 Although this is an important area in Cameroon, it is a tiny part of the country, Africa, and the world. What makes this study unique and of interest to scientists worldwide?

L186 Ideally, the veterinarian should have changed gloves between each teat, not just each cow.

L223 Please report the precise statistical models used for these analyses.

L232 Describe how the cows’ teats were cleaned before milking and whether their teats were dipped with disinfectants after milking. Also, did the milkers wear gloves and change gloves between cows? If none of these procedures were followed, then the levels of infection would be greater than if recommended milking procedures were used. Was the milking procedure recorded for each farm?

L234 What was the average lactation number for the cows? Among these herds, what was the average stage of lactation at sampling?

L252 ….13 out of 1644 quarters (2.2%) were….

L253-254 ….were blind, and 5.8% of the 1608 functional quarters (within 33 cows) displayed clinical signs characterized by watery milk or clots, flakes or blood.

L261 This entire section and tables are not useful or informative. There were too few cows per Division to be meaningful. The information may be useful for local farmers, but it is not useful for the scientific community at large.

L280 The term “subclinical mastitis” is not clearly defined by NMC or other organizations. Somatic cell count of milk that is acceptable for processing for human consumption differs among countries and even among processors within countries. Clearly, cows with extended periods of SCC above 100,000 produce less milk, but these cows are not classified as having mastitis unless the milk is abnormal or the udder is swollen, produces bloody milk or milk clots. It is more meaningful to look at the average SCC for the herd, but in this study, herd sizes are small, and a single cow can affect the average for the entire herd.

L280-onward Number of cows in each region is too small to be meaningful to the scientific community. This is useful information for the regions, but it is not useful for dairy farms elsewhere, because regions may differ in rates of infection.

L310 This section is useful and should be retained.

L350 This section is useful and should be retained.

L382 This section can be condensed to key points without making excessive comparisons among different countries. Also, include reports from dairy regions outside of Africa.
