# Supplementary material for: Prevalence and risk factors of coliform-associated mastitis and antibiotic resistance of coliforms from lactating dairy cows in North West Cameroon
Source: PLoS One. 2022 Jul 26;17(7):e0268247. doi: 10.1371/journal.pone.0268247 (PMC9321367; doi:10.1371/journal.pone.0268247)
Supplement: Attachment — Submitted filename: Response to Reviewers.docx [file pone.0268247.s003.docx]

**Point-by-point response letter**

Dear Editor,

We appreciate your comments and those of the reviewers. We hope we have provided satisfactory responses/amendments to all the comments. The Point-by-point responses/ amendments are presented below.

**Editor’s comments**

**Editor:** We invite you to submit a revised version of the manuscript that addresses the points raised during the review process.

**Response:** We have submitted a revised version of the manuscript addressing the points raised during the review process. Thank you for this opportunity. Hope we have addressed the points satisfactorily.

**…………………………………………………………………………………………………**

**Editor:** Please include the following items when submitting your revised manuscript:

- A rebuttal letter that responds to each point raised by the academic editor and reviewer(s). You should upload this letter as a separate file labeled 'Response to Reviewers'.
- A marked-up copy of your manuscript that highlights changes made to the original version. You should upload this as a separate file labeled 'Revised Manuscript with Track Changes'.
- An unmarked version of your revised paper without tracked changes. You should upload this as a separate file labeled 'Manuscript'.

**Response:** We have submitted all items requested above.

**…………………………………………………………………………………………………**

**Editor:** Please review your reference list to ensure that it is complete and correct. If you have cited papers that have been retracted, please include the rationale for doing so in the manuscript text, or remove these references and replace them with relevant current references. Any changes to the reference list should be mentioned in the rebuttal letter that accompanies your revised manuscript. If you need to cite a retracted article, indicate the article’s retracted status in the References list and also include a citation and full reference for the retraction notice.

**Response:** We have reviewed the reference list to ensure it is complete and correct.

**………………………………………………………………………………………………………**

**Editor:** While revising your submission, please upload your figure files to the Preflight Analysis and Conversion Engine (PACE) digital diagnostic tool, <https://pacev2.apexcovantage.com/>.

**Response:** We have uploaded figure files to the PACE digital diagnostic tool to ensure they meet PLOS requirements.

**………………………………………………………………………………………………………**

**Reviewer #3 comments**

**Reviewer #3:** I only have minor comments for this version.
Lines 477-490: when it comes to the discussion of percent resistant isolates, I think the authors should be much more cautious in their statements. What is the meaning of 100% resistant isolates for a particular antibiotic when only one or two strains of a species have been isolated? Not much. If read as such, one could take as a general message that all *Serratia* spp. or *Citrobacter freundii* isolated from milk are resistant to tetracycline. Drawing such a conclusion from 1 or 2 isolates is really misleading. I think the authors should somehow nuance their remarks and focus on species for which they have at least 10 isolates.

**Response:** We have revised this. *Serratia* spp. and *Citrobacter freundii* with 4 and 2 isolates respectively have been excluded in the discussion. See L477-488

**………………………………………………………………………………………………………**
**Reviewer #3:** Line 475: the statement "Thus, it is important to isolate the coliform mastitis pathogen and perform an antibiotic susceptibility test before any antibiotic therapy." is not always realistic when it comes to application on the field. Very often, when a clinical mastitis case is detected, antibiotics are used without any prior identification of the pathogen. I am not saying it is not important to follow antibiotic resistance of mastitis pathogens, I'm rather implying that it not easily feasible in the field and that it is more a question for surveillance authorities. This statement should be somehow modified.

**Response:** Thank you, this statement has been revised. See L475-476

**………………………………………………………………………………………………………**
